# Reach, Engagement, and Acceptability of a Subclinical Telehealth Service for Spanish-Speaking Adults: Retrospective Mixed Methods Pilot Study

**DOI:** 10.2196/80026

**Published:** 2025-12-31

**Authors:** Marvyn R Arévalo Avalos, Julio Fu, Adrian Aguilera, Luis Suarez

**Affiliations:** 1 School of Social Welfare, University of California, Berkeley, 120 Haviland Hall, Berkeley, CA, 94720, United States, 1 510-255-1767; 2 Sanarai, Chicago, IL, United States; 3 Department of Psychiatry and Behavioral Sciences, University of California, San Francisco, San Francisco, CA, United States

**Keywords:** digital health, Latino/x, mixed methods, Spanish-language mental health care, telehealth services

## Abstract

**Background:**

There is a gap in mental health care among Latino/x and Spanish-speaking communities, and the care that is available is often difficult to access, lacks cultural nuance, and results in low engagement and satisfaction.

**Objective:**

The study aimed to evaluate the reach, adoption, and acceptability of digital Spanish-language psychosocial and emotional wellness services among Latino/x adults offered by the digital health company Sanarai.

**Methods:**

Data included in this study were collected between August 2020 and September 2024 by Sanarai as part of its ongoing services. Quantitative data sources included individual customers’ appointment data, individual session payment data, and customer satisfaction data. Qualitative data were obtained from transcribed notes of telephone or video-based user interviews conducted by Sanarai staff between August 2020 and May 2024.

**Results:**

Between August 2020 and September 2024, Sanarai served 6163 users (n=3662, 59.42% women participants) across all 50 US states, with the highest concentration of participants in Texas and California. Results showed 94% (n=5793) of users scheduled a first appointment within 1 week, with 43% (n=2650) doing so within 1 day. Over 62.60% (n=3858) of participants engaged in two or more sessions, attending an average of 8.94 (SD 13) sessions over 110 days (SD 169). The platform delivered a total of 36,858 appointments, including individual and couples sessions. Only 22.47% (n=1385) of users responded to a customer satisfaction survey for a total of 2287 distinct responses; among this subgroup, session satisfaction was high with an average satisfaction rating of 4.88 out of 5.0 (SD 0.49) and a Net Promoter Score of +85. Nearly all responses (n=2174, 95.06%) expressed intent to schedule another session, but these results should be interpreted with caution, given the low response rate. Qualitative interviews with 30 users (n=21, 70% women) revealed a diverse user base. Many users reported prior mental health service experiences, while one-third were new to care. Participants cited cost, cultural fit, language access, and convenience as key reasons for choosing Sanarai over local services. Users highlighted the platform’s affordability, scheduling flexibility, and provider professionalism as central to their positive experiences.

**Conclusions:**

These findings underscore the value of culturally responsive, accessible online mental health care for Spanish-speaking communities.

## Introduction

Latino/x individuals, who represent 20% of the US population [1], experienced a high level of unmet need for mental health services [2]. Estimates suggested that nearly 1 in 2 (41%‐49%) of Latino/x people with a perceived need for mental health services also experienced unmet mental health care [3-5]. Notably, 58% of foreign-born Latino/x adults and 12% of the US-born adults preferred a Spanish-speaking health care provider or a Latino/x health care provider (47% among foreign-born and 20% among the US-born Latino/x) [6]. However, according to the American Psychological Association, only 5% of the psychologist workforce offered Spanish-language services [7], and only 5% to 6% of the mental health counselor and psychologist workforce identified as Latino/x [7,8]. There was a critical need for services and resources that addressed disparities in mental health care among Latino/x adults in the United States [9-11]. Cost, stigma, and language access were often cited as barriers to care. The cost of mental health services in the United States poses a barrier for uninsured and underinsured communities [11], which is characterized by many Spanish-speaking Latino/x individuals. Stigma was also cited as a possible factor deterring individuals from seeking care due to not wanting to feel ostracized, identified as mentally ill, or perceptions of mental health needs as weakness [12,13]. The large unmet mental health needs of vulnerable populations [4,5,14] are detrimental to individuals and families. There was an urgent need to develop and evaluate solutions that were accessible and culturally relevant for Latino/x and Spanish-speaking communities.

The rapidly growing field of digital mental health, particularly online therapy or digital mental health coaching, has the potential to meet the needs of vulnerable populations. Digital solutions also tended to be more private and could help reduce stigma. However, many of these digital efforts were limited in reaching and engaging Spanish-speaking and Latino/x populations. Among Latino/x adults, acceptability and engagement with digital mental health interventions were often low [15,16], thus negatively impacting the potential benefits of digital mental health solutions for this population. Reasons for low engagement and satisfaction were that most digital health solutions were not linguistically or culturally congruent, particularly for Spanish-speaking adults, and were difficult for Latino/x adults to access (eg, cost, time, and availability) [11,17]. The COVID-19 pandemic gave rise to the rapid expansion and adoption of telehealth mental health services across the United States [18], but these gains were not observed across all demographic groups. For example, mental health treatment centers in communities with high proportions of Latino/x residents were twice as *unlikely* to adopt telehealth services relative to communities with smaller proportions of Latino/x residents [19]. These discrepancies in the adoption of telehealth services have contributed to greater disparities in unmet mental health needs among Latino/x adults, particularly those who preferred Spanish-language services. In addition, a national evaluation of digital health technologies among Spanish speakers in the United States found that digital literacy, limited availability and appropriateness of information in Spanish, and the lack of Spanish-speaking interpreters limited patients’ adoption of online health portals and telehealth services; whereas, facilitators of digital technology adoption included access to interpreters and culturally and linguistically tailored Spanish-language materials and health care providers [20]. Collectively, these results indicated that the lack of linguistically and culturally appropriate services and providers was a significant barrier to accessing care among Spanish-speaking communities.

One way to address the shortage of Spanish-speaking providers in the United States was to connect with mental health providers in predominantly Spanish-speaking countries via digital health technologies. For example, private sector digital health companies, such as Sanarai, connected Spanish-speaking clients in the United States to licensed mental health professionals in Latin America via telehealth with the goal of providing linguistically and culturally competent subclinical mental health services [21,22]. These types of programs using providers from Latin America for mental health support have not been formally evaluated, but there was evidence to suggest that these providers could fill existing gaps in care. For instance, evidence from primary care programs, such as the Licensed Physicians from Mexico Pilot Program (LPMPP) implemented in California in 2002, suggested that working with providers abroad could help address existing domestic gaps. The LPMPP was approved with the goal of taking 30 licensed physicians from Mexico to provide medical care to underserved Latino/x and Spanish-speaking communities at nonprofit community health centers throughout the state to address shortages of health care providers and to increase linguistically and culturally competent care [23]. An evaluation of the LPMPP indicated that the program met the intended goals of improving access to linguistically and culturally competent care and resulted in high acceptability of the LPMPP physicians among patients, particularly among monolingual Spanish-speaking patients [23]. The results of this program were promising and provided some evidence of the benefits of addressing gaps in care and the shortage of Spanish-speaking mental health professionals with providers from Spanish-speaking countries.

As noted earlier, private sector companies were bridging gaps in access to linguistically and culturally grounded mental health care for Latinos/x and Spanish-speaking communities. Although private companies were often not set up to test specific hypotheses, it was important to apply implementation frameworks to understand if interventions were acceptable, feasible, and addressing needs. Acceptability and engagement were two important and interrelated implementation constructs that were associated with the effectiveness of digital mental health interventions [24-27]. Results from existing research indicate that acceptability impacted engagement, and vice versa, and that both constructs were influenced by the user, intervention, and technology factors [24,27]. For example, facilitators include user beliefs toward technology and mental health, perceived fit and usefulness of the intervention, ease of integration of the digital solutions into one’s life (eg, cost and availability), and privacy and confidentiality of the digital tools [24,26,28,29]. Much of the recent extant digital mental health research focused on the use of smartphone apps or other technology-delivered, asynchronous, self-paced interventions and was limited in its inclusion of Spanish-speaking populations [25,28,30,31]. Thus, there was a need to evaluate whether telehealth-delivered mental health supportive services for Spanish-speaking adults were acceptable, engaging, and potentially viable digital health solutions to address gaps in care for this vulnerable population.

The purpose of this mixed methods retrospective pilot study was to evaluate and describe the implementation of a telehealth-delivered, Spanish-language, psychosocial and emotional support service among Spanish-speaking adults. The mixed methods approach was useful in health services research [32] as the quantitative data helped assess the magnitude of impact or outcomes (eg, reach and engagement) while the qualitative data helped understand the process and content of use. The inclusion of rich interview data provided a more comprehensive understanding of the multidimensional facets of intervention acceptability and engagement, which might not otherwise be obtained via survey data. This study examined the reach, engagement, and acceptability of the Spanish-language telehealth services by analyzing quantitative and qualitative data gathered via the routine services provided by the digital health company Sanarai. In addition, the results of this study aimed to provide evidence regarding the feasibility of leveraging mental health providers from Spanish-speaking countries to meet existing gaps in care in linguistically and culturally appropriate mental health services for Latino/x and Spanish-speaking adults.

## Methods

### Sanarai

Sanarai is a digital health company that arranges for the delivery of psychosocial and emotional supportive services to adults (aged ≥18 y) via telehealth technologies from licensed Spanish-speaking professionals from Latin America to Spanish-speaking adults located in the United States and other countries. These services are primarily provided direct-to-consumers and target Latino/x and Spanish-speaking populations residing in the United States with the goal of increasing access to high-quality, culturally congruent, and affordable subclinical mental health supportive services. The company services include: advice related to general mental health and wellness; life coaching and recommendations for setting personal goals; emotional support related to interpersonal relationships, sexuality, and gender; and emotional support related to feelings of anxiousness, stress, and depression. Sanarai addresses the subclinical population, emphasizing support for individuals dealing with challenges that may not meet the clinical threshold but still significantly impact their well-being. The focus of the services is to provide early intervention and preventive support. The company was founded in 2020.

Sanarai has developed a simple workflow to onboard users into its platform. First, users can browse the up-to-date provider database found on the Sanarai website to select the provider of their choice (step 1) and choose their desired appointment time (step 2). Then, users provide basic contact and payment information (step 3) to book the appointment, which is confirmed via email and conducted via Zoom (Zoom Communications, Inc; step 4). Users have the flexibility of changing their provider at any time in the process.

Sanarai users may sign up for an introductory 30-minute initial consultation or a full 50-minute session, either as an individual or as a couple. All sessions are conducted via live video calls on the video platform, Zoom. At the time of this evaluation, the initial consultation was priced at US $20.00 and served as an opportunity for the user to get to know their provider and their working style. The individual and couples’ sessions, US $49.00 and US $59.00, respectively, are focused on addressing the psychosocial and emotional wellness needs of the user. Sanarai’s providers are licensed mental health professionals from Latin America with at least a master’s degree and a minimum of 5 years of clinical experience. However, Sanarai notifies its users that despite the providers’ educational background and clinical expertise, Sanarai’s services are not considered psychiatric, psychological, or mental health therapy services as traditionally provided by a licensed mental health provider in the United States. Thus, Sanarai providers do not diagnose, prescribe medication, or accept health insurance. As needed, Sanarai will provide resources and referrals to users who need a higher level of care or are seeking services related to mental health diagnoses or medication management.

### Data Sources and Analysis

Sanarai has routinely collected quantitative and qualitative data as part of its ongoing services. Data included in this study were collected between August 2020 and September 2024. Quantitative data sources included three sources: (1) individual customers’ appointment data, (2) individual sessions payment data, and (3) customer satisfaction data. Specifically, all appointment and user data (1) were sourced directly from the booking platform, Acuity Scheduling, via automatic export. This system was consistently used throughout the entire evaluation period, ensuring uniformity. This appointment data included the date and time of each appointment, the type of appointment, the price and amount paid for each appointment, and whether the user was referred via an organizational partnership. Users’ first name was used to generate a gender variable consistent with Spanish-language name norms (ie, first names ending with the vowels “a” or “e” were coded as “Woman participants,” those ending with “o” were coded as “Man participants,” and a manual review was done to double check the coding). Payment data (2) was used to extract the user’s zip code, if available. These quantitative data served as the basis to analyze reach and adoption by examining trends in users’ engagement and session data. In addition, after each individual session, Sanarai automatically emailed users inviting them to participate in a brief web-based satisfaction survey (3) using a standardized Google Form, which included questions about satisfaction with services, willingness to recommend the services, and the Net Promoter Score measure. These data provided a snapshot of the acceptability of the service.

Quantitative data analysis was conducted using R software (R Foundation for Statistical Computing) [33] and included computing descriptive statistics for the number of users, the number and types of sessions attended, session engagement metrics, and the geographic distribution of users across the continental United States. Though this study was focused on a description of implementation outcomes and not hypothesis testing, exploratory analysis was conducted to examine usage patterns by gender and by time to schedule the first appointment.

Qualitative data were obtained from transcribed notes of telephone or video-based user interviews conducted by Sanarai staff (3 members of the executive team) between August 2020 and May 2024. The primary goal of these interviews was internal product improvement, not formal research, yet these interviews were structured and used a questionnaire based on the “Jobs To Be Done” framework. The “Jobs To Be Done” framework focused on understanding the motivations and decisions that drive a user to select a product or service to accomplish their goals or meet their needs (aka meet the requirements of the job they want done) [34]. The interviews focused on understanding the user experiences, motivations, and preferences for seeking mental health services with Sanarai. Participants were recruited via generalized outreach to the entire user base on a quarterly basis. Participation in these interviews was based on 100% self-selection, and everyone who expressed interest was interviewed. At times, the company prioritized interviewing “super users” (high session count) or users who had recently stopped seeking services in order to understand specific use patterns. Users who opted in to participate in these interviews were offered a discount of up to 15% on their next session. These qualitative data served as the basis to analyze acceptability.

We examined the user interview transcript and conducted reflexive thematic analysis (TA) [35,36] to examine users’ attitudes and experiences with seeking and accessing mental health services, including services provided by Sanarai. The reflexive TA was conducted by the first author, who began by reviewing the transcripts and independently coding the data. Codes were generated based on an inductive data-driven and semantically focused approach [36] with the goal of summarizing patterns in the data via themes that best represented the beliefs and experiences of the participants. A key concept in reflexive TA was that themes emerge at the intersection of the data and the researcher’s subjectivity and research skills [36]. Thus, to provide an extra layer of validation of the qualitative findings, the codes, themes, and narrative presentation of the qualitative results were reviewed and approved by the second and fourth authors, who had more familiarity with the interview process and qualitative interviews.

### Ethical Considerations

This study was reviewed by the University of California, Berkeley Institutional Review Board (IRB #2024-08-17735) and granted exempt status as it satisfies the federal and UC Berkeley requirements under category 70: research that involves no greater than minimal risk to subjects. The data used in this study were collected by Sanarai, as part of enrollment in its services, users provide broad consent to the potential use of their data for program evaluations and secondary analysis. Data were not fully anonymized, as the data sources included a unique Sanarai ID comprised of the email address the participant used to enroll in the services. To safeguard participant information, data were shared directly between Sanarai and the first author and stored under university-sponsored password-protected accounts. Data were accessed under a data transfer agreement between the University of California, Berkeley and Sanarai. Participants who completed the user interviews were offered a discount of up to 15% for their next session. No additional participant compensation was provided. The study’s conceptualization, analysis, and interpretations were conducted independently by the first author.

## Results

### Reach

**Figure 1.**
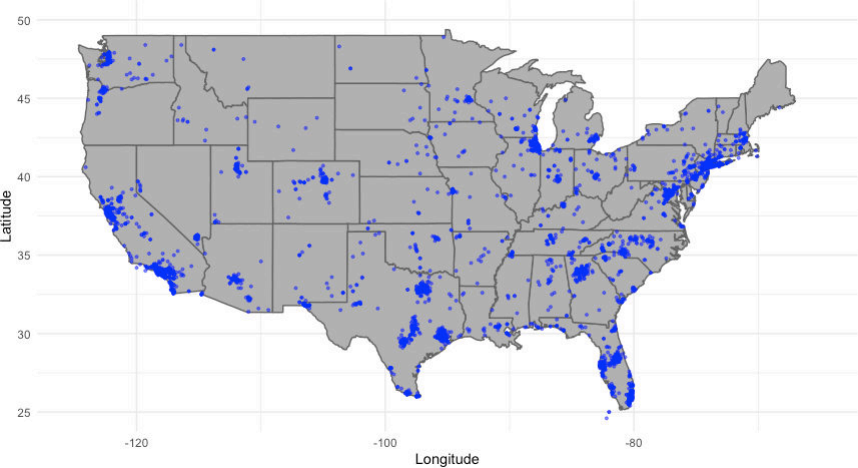
Participant locations in the continental United States. Each blue dot represented an approximate location for each participant. Location estimates were based on available participant zip code data.

**Figure 2.**
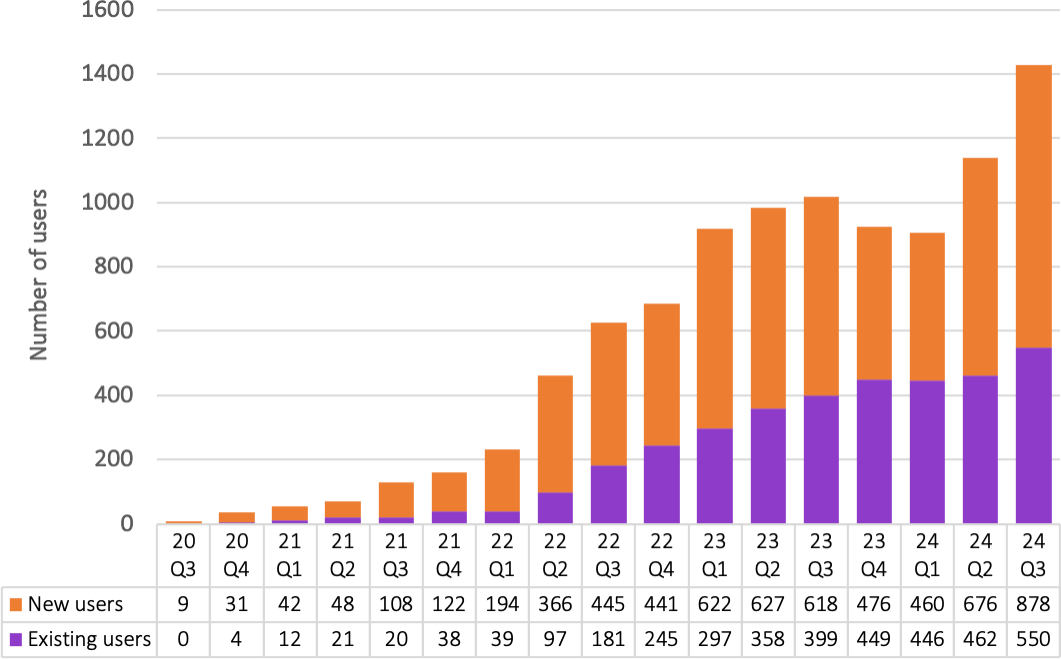
Quarterly trends of Sanarai users. This stacked bar graph represents the total numbers of active users in the online telehealth service, Sanarai, for each calendar quarter starting from quarter 3: July 2020-September 2020 (20 Q3) through quarter 3: July 2024-September 2024 (24 Q3). The orange (top) bars corresponded to new users in the service, the purple (bottom) bars corresponded to existing users. The table below the bar graph includes the counts for each new and existing user by quarter.

During the 4-year period between August 2020 and September 2024, Sanarai provided psychosocial and emotional wellness support services to a total of 6163 users. The majority of users were women (n=3662, 59.4%), and the remaining were men (n=2501, 40.6%). In terms of geographic region, payment data indicated that 65.7% (n=4049) of users made payments using a credit card obtained in the United States; 6.27% (n=387) made payments using a credit card obtained outside of the United States. For the remaining 28.02% (n=1727) of users, their geographic region was unknown as the payment data did not include country or zip code information. Within the US-based sample, 97.06% (n=3930) of the users had available zip code data, which were used to estimate participant locations within the United States (Figure 1). In sum, all 50 states of the United States, the District of Columbia, and Puerto Rico were represented in the sample, yet 55% of the users were concentrated in just 5 states: Texas (n=621, 15.34%), California (n=567, 14%), Florida (n=539, 13.31%), New York (n=246, 6.08%), and New Jersey (n=190, 4.69%). All other states represented 43.64% (n=1767) of the users, and 2.94% (n=119) were unknown. Sanarai users were reached via online advertisements, partnerships with community-based organizations, and word of mouth (recommendations by family and friends). During this evaluation period, Sanarai’s user base and number of active users grew an average of 47% quarter over quarter (Figure 2).

### Engagement

Among the 6163 users served by Sanarai, the first point of contact for 53.33% (n=3287) was an initial consultation, and the rest (n=2876, 46.67%) began with a full session. In terms of appointment availability, 43% (n=2650) of users scheduled their first appointment within 1 day or less, and an additional 30% (n=1849) scheduled within 2 to 3 days. Overall, the vast majority of users (n=5793, 94%) scheduled a first appointment within 7 days, and a minority scheduled their first appointment after more than 7 days (n=370, 6%).

Regarding engagement pattern and service use types, 36.26% (n=2235) of users attended 1 appointment only (either a consultation or 1 single full session), and 1.14% (n=70) had consultations without a single full session; whereas 62.6% (n=3858) of users engaged with Sanarai services at least two or more times via a combination of consultation and sessions or sessions alone (Table 1). Among this subsample of users with greater than or equal to 2 sessions of any type, we calculated the total number of sessions attended, days between sessions, and time between first and last session. Full descriptive statistics for these engagement metrics are reported in Table 2. In sum, users attended an average of 8.94 sessions, with an average of 12.4 days between sessions, and remained engaged for an average of 110 days. Finally, during the evaluation period, Sanarai provided a total of 36,858 appointments—including individual consultations (n=3563, 9.67%), individual sessions (n=31 905, 86.6%), couples sessions (n=1179, 3.2%), and couples initial consultations (n=211, 0.57%). This represented approximately 768 appointments per month.

**Table 1.** Engagement of Sanarai users.

Engagement	Value, n (%)
First appointment type	
Initial consultation	3104 (50.37)
Couples initial consultation	183 (2.97)
Individual session	2643 (42.88)
Couples session	233 (3.78)
Service use types	
Consultations only	1214 (19.70)
Single session	1091 (17.70)
Multiple sessions	1700 (27.58)
Consultation + sessions	2158 (35.02)

**Table 2.** Engagement metrics.

	Median (IQR)	Mean (SD)	Minimum	Maximum	SE (95% CI)
Total number of sessions	5 (3-9)	8.94 (13.02)	2	236	0.21 (8.53-9.35)
Days between sessions	7 (4.50-11.50)	12.40 (20.96)	0	521	0.34 (11.74-13.06)
Days engaged	40 (14-121)	110.49 (168.95)	0	1445	2.72 (105.15-115.82)
Time to schedule first appointment (any type)	2 (1-4)	2.80 (2.62)	1	27	0.04 (2.72-2.88)

To further contextualize engagement patterns, we conducted two exploratory analyses. First, we conducted an independent samples 2-tailed *t* test to examine whether the total number of sessions and the total number of days engaged differed by gender. The results indicated that there were no statistically significant differences in total number of sessions between women participants (mean 8.99, SD 12.75) and men participants (mean 8.86, SD 13.41; *t*_3338.6_=0.29; 95% CI −0.72 to 0.97; *P*=.77). Similarly, we did not observe any statistically significant differences in the number of days engaged (women participants: mean 114.27, SD 169.89; men participants: mean 105.15, SD 167.57; *t*_3476.6_=1.66; 95% CI –1.67 to 19.92; *P*=.10). Finally, we conducted a correlation between time to schedule first appointment and total number of sessions to examine if prompt services were associated with greater intervention engagement and found a very small and weak negative correlation (*r*=−0.038, 95% CI −0.063 to −0.013).

### Acceptability

#### Overview

Sanarai’s session satisfaction survey was completed by 22.47% (n=1385) of its users, resulting in 2287 distinct responses. These data showed a Net Promoter Score of +85, which indicates that a large portion of respondents were likely to recommend the services to friends and family. In addition, there was an average session satisfaction rating of 4.88 (SD 0.49; rating 1=very bad and 5=very good), and 95.06% (n=2174) of responses indicated “Yes” to scheduling a new session. These data provided a snapshot of high acceptability for Sanarai services among this user base, but this should be interpreted with caution given the low response rate.

Qualitative data (user interview data) were analyzed from 30 participants. These data were collected between August 3, 2020, and May 29, 2024. There was an overrepresentation of women participants (n=21, 70%) versus men participants (n=9, 30%). Participants had an average age of 30.54 (SD 6.81; range 19‐46) years. Participants were diverse in terms of country of origin, with Mexico representing 43.33% (n=13) of the sample. The US state of residence distribution was similar to the full sample (Table 3), and the length of time residing in their state ranged from 2 months to 30 years (mean 6.73, SD 6.60 y). One-third of users (n=10, 33.33%) reported not having any past mental health service experiences. The remaining users (n=20, 66.67%) reported having prior experience with mental health services, which varied from long-term exposure (n=8), recent exposure only (n=8), or exposure in one’s home country (n=5).

**Table 3.** Demographics of user interviews.

Characteristic	Value, n (%)
Gender	
Woman	21 (70)
Man	9 (30)
Country of origin	
Mexico	13 (43.33)
United States	4 (13.33)
Venezuela	4 (13.33)
Dominican Republic	3 (10)
Other^a^	6 (20)
State residing	
California	5 (16.67)
New York	5 (16.67)
Florida	3 (10)
Texas	3 (10)
Other^b^	14 (46.67)

a Ecuador, Guatemala, Colombia, Puerto Rico, and Spain.

b Georgia, North Carolina, Connecticut, Illinois, Massachusetts, Michigan, Minnesota, New Jersey, New Mexico, Pennsylvania, Tennessee, and Washington State.

#### Reflexive TA Themes

##### Reasons for Seeking Services

Users reported a wide range of reasons for which they were seeking mental health support. These reasons included a history of mental health concerns, general mental health stressors, transitions related to stress, family-related stress, and self-growth. Several users reported seeking support to manage symptoms related to depression, anxiety, binge eating, insomnia, or other undisclosed history of mental health issues. On the other hand, some users were seeking support to address general mental health stressors, increased emotionality and vulnerability, grief, or acute life stressors. In terms of transition-related stress, users identified immigration-related stress, missing family or their home country, coping with the COVID-19 pandemic, and loneliness as reasons for needing support. Family stressors included marital and relationship conflicts, concerns about supporting children’s mental health, and concerns about one’s family. Finally, a minority of users reported seeking services as a form of personal improvement and self-growth.

##### Use of Local Resources

In discussing participants' decision to seek online services, users described their experiences and beliefs about seeking local, in-person resources to meet their needs. Participants discussed four main themes that presented as barriers to using local resources, which included cost, accessibility, cultural fit, and modality fit. First, most participants reported that local mental health services were too expensive; that mental health services were not covered under their health insurance; or that even if those services were included, the co-pays and deductibles would be comparable to Sanarai’s service pricing and, thus, considered cost a significant barrier to seeking mental health services in their local communities.

Accessibility was another prominent issue reported by participants, which included barriers with transportation to in-person services, difficulty navigating the US health care system, lack of available providers, and long wait times to receive services. A third barrier to using local resources was related to the cultural fit of services, with users reporting that despite looking for help, they were unable to find Spanish-speaking language providers or culturally competent care. Finally, users reported issues with a lack of modality fit, meaning that when they sought services for mental health, they were referred to psychiatry, which focused too much on medication prescription when they preferred talk therapy, or that even when providers advertised having available in-person appointments, they would only accept patients for video-based appointments.

##### Value of Sanarai or Virtual Mental Health Support

Participants reported that Sanarai and online mental health support service value was driven by its ability to overcome barriers related to accessing local services. Specifically, many users endorsed the ability to receive Spanish-language services and culturally congruent care from a trustworthy and professional source as a main driver for seeking online services via Sanarai. Accessibility was another important consideration for users, as they stated they liked the ease of scheduling sessions, flexibility in scheduling, ease of making payments, and choice about selecting or switching their provider.

Cost was also cited as a reason for choosing Sanarai versus other resources. Although to a lesser extent than the previous themes, some users reported that Sanarai’s services were affordable and that receiving a free initial consult motivated them to engage in services. For example, some users noted issues related to affordability and health insurance. Finally, a minority of users endorsed a variety of other topics, including finding the website attractive and professional, having reliable technology to conduct sessions, and appreciating the privacy and confidentiality of Sanarai’s services, specifically this service not being connected to a user’s medical record.

##### Experience With Sanarai Providers

Users reported choosing their specific provider because their online profile fit the patient’s needs. For example, some users identified a provider who met their demographic or identity preferences (eg, ethnic background, gender, and religion). Others thought the providers’ years of experience and areas of expertise or focus were a good fit to address their concerns. Users also reported feeling comfortable communicating with their providers due to their level of professionalism, empathy, helpfulness, and understanding expressed throughout sessions. However, a minority of users reported having mixed reactions toward their provider and reported feeling as if their provider was not as attentive or providing the right type of support. These users acknowledged that when they were not feeling comfortable with their provider, they knew they were able to switch providers, yet some chose to terminate services instead.

## Discussion

### Principal Findings

This study evaluated the reach, engagement, and acceptability of a telehealth Spanish-language psychosocial and emotional wellness service offered by a digital health company, Sanarai. The service delivery model focused on connecting users to Spanish-speaking licensed mental health providers from Mexico and Argentina to receive subclinical mental health supportive services via telehealth. The results of this mixed methods evaluation suggest that Sanarai services are accessible and acceptable as evidenced by the reach of the services to over 6000 users across the United States, an average duration of 9 sessions attended per user, and some preliminary indication of high satisfaction rates. Factors that may contribute to Sanarai’s model acceptability include providing prompt (next-day session availability), relatively affordable rates compared to market rates in the United States (US $49-$59 for individual and couples’ sessions), offering culturally and linguistically congruent mental health services for Latino/x adults, and having an accessible and easy-to-use technology platform.

A central goal of the Sanarai model of mental health support was to increase the availability of language-concordant mental health providers for Spanish-speaking adults. The geographic reach of Sanarai’s services is representative of the widespread need for professional mental health care services in the United States. According to the Health Resources and Services Administration, there is a significant shortage of mental health professionals at the national level, as only 26.4% of the mental health professional workforce needs were met as of March 31, 2025 [37]. According to the report, in the top 5 US states where Sanarai had the highest user enrollment, the percent of needs met were 15.6% (New York), 22.4% (California), 23.7% (Florida), 31.3% (Texas), and 52.7% (New Jersey) [37], indicating an ongoing significant need for additional mental health professionals. Further, between 2014 and 2019, there was a 17.8% decrease in facilities that offered mental health treatment in Spanish despite a 4.5% growth in the Latino/x population during the same time period [38]. The importance of having access to Spanish-speaking health care providers cannot be overstated, as current estimates suggest only 5% to 6% of providers identify as Latino/x and were able to provide Spanish-language services [7,8], and many Latino/x people preferred to receive health care services in Spanish, particularly when discussing emotional concerns [6,39,40].

In terms of availability, Sanarai users waited on average 2.87 days to meet with their provider. The availability of Sanarai’s providers is drastically different from the availability of psychologists in the United States. According to a 2023 national survey by the American Psychological Association, more than half of psychologists (56%) reported having no openings for new patients or having a waitlist of 3 months or more [41]. The risk of long wait times to receive services may result in a patient’s worsening mental health [42] and greater health care expenditures, or patients may lose interest in engaging in services despite having an ongoing need. In this study, we did not find a significant association between appointment waiting time and total engagement with services. Sanarai users in this study attended an average of 9 sessions. Comparatively, other studies have shown that Latino/x individuals attend an average of 5 sessions for remote telephone-based services [43] and 8 sessions for in-person mental health care [15]. Finally, women typically endorse more positive health-seeking attitudes for mental health issues than men [44], are more likely to report needing and accessing mental health services [45], and are more likely to engage with digital mental health interventions [24]; however, in this study, we did not find any statistically significant differences in session engagement or duration of treatment by gender, suggesting that both men and women accessed and engaged with Sanarai at similar rates. Examining additional demographic variables, such as age and educational background, can further help explain patterns in service use among diverse segments of the population.

The qualitative data provides a rich description of the various reasons why Sanarai users engaged with the services and helps contextualize the quantitative findings. Contrary to the quantitative data in which there was a more balanced breakdown of gender (60% women participants and 40% men participants), the respondents of the qualitative surveys were predominantly women (70%); thus, these results should be interpreted with caution due to the potential risk of bias and limited generalization. The respondents’ reasons for seeking services were varied in intensity and ranged from managing symptoms related to mental health concerns to addressing situational stressors and self-growth. Severity of symptoms has been associated with greater engagement with digital mental health interventions [9,24] and may partly explain the engagement rates observed in this study. Users noted a wide range of barriers to accessing in-person mental health services within their communities. Many of the issues raised were also well documented in the extant literature, including barriers due to language fluency, cultural differences between patient and provider, lack of access to low-cost mental health services, and perceived lower quality of mental health care [5,45,46]. For the participants in this study, these barriers may have been the precursor to engagement and acceptability of digital mental health services.

Acceptability of digital mental health services in general, and Sanarai’s services specifically, can be understood within the multifaceted framework of health care intervention acceptability [26,27]. Specifically, users endorsed ease of access, scheduling, technology navigation, and availability of providers (eg, low burden of engagement) as a significant facilitator to engaging with Sanarai. Similarly, the users perceived the services to be affordable and a good fit given their cultural values, goals, and language preference, and perceived the providers as trustworthy and helpful (ie, concurrent acceptability), which are all facilitators associated with increasing acceptability, engagement, and effectiveness of digital mental health interventions [24,26,27,39,40]. These findings provided evidence for the value of leveraging mental health providers from the Spanish-speaking countries to deliver mental health supportive services to Spanish-speaking adults in the United States. In addition, it is unclear how the acceptability of the service would impact clinical outcomes; thus, future research may include an outcomes-based evaluation or a randomized clinical trial design.

This study had several limitations. First, the data were primarily descriptive, based on users from one private telehealth company, and did not have a comparison group. These limitations limit generalizability, as it was possible that the sample included in this research was not representative of the broader Spanish-speaking populations. Also, due to the limited availability of demographic data collected via the appointment data, we were unable to examine the representativeness of the sample and how known factors such as income or health insurance status impacted users’ participation with Sanarai services. Similarly, at the time of this evaluation, Sanarai was beginning to collect clinical effectiveness data; thus, we were not able to assess the clinical impact of these online services on users’ mental health outcomes and how these outcomes are related to engagement and acceptability. This limitation remained an area of priority for future work. Regarding acceptability, though the user satisfaction survey was shared regularly with all users active in the service—less than 25% of the total sample participated in this survey—potentially limiting the generalizability of these findings to the entire sample and inflating positive findings as a result of self-selection bias. In addition, the results focused on acceptability were based on the qualitative data analysis, which included an overrepresentation of women relative to men and thus did not generalize. Similarly, the user interviews were conducted by company staff, and despite using a standardized protocol, it was possible that users responded with bias. Areas for future directions included collecting clinical outcomes data; increasing the collection of demographic data to facilitate greater exploration of reach, engagement, and acceptability patterns; increasing representativeness of samples; and increasing response rates to satisfaction surveys.

### Conclusions

Overall, Sanarai’s model appears to be meeting a need for mental health services for Spanish-speaking Latino/x communities by delivering accessible, prompt, and linguistically and culturally congruent mental health supportive services.

## Abbreviations

LPMPP: Licensed Physicians from Mexico Pilot Program
TA: thematic analysis
